# Comparative Study of Polymer-Modified Copper Oxide Electrochemical Sensors: Stability and Performance Analysis

**DOI:** 10.3390/s24165290

**Published:** 2024-08-15

**Authors:** Andrzej Baziak, Anna Kusior

**Affiliations:** 1 Faculty of Physics and Applied Computer Science, AGH University of Krakow, Al. Mickiewicza 30, 30-059 Krakow, Poland; abaziak@student.agh.edu.pl; 2 Faculty of Materials Sciences and Ceramics, AGH University of Krakow, Al. Mickiewicza 30, 30-059 Krakow, Poland

**Keywords:** binding agent, electrochemical sensor, drop casting, Nafion, PVP, HPC, α-terpineol, CuO, electrostability

## Abstract

The effectiveness of copper oxide-modified electrochemical sensors using different polymers is being studied. The commercial powder was sonicated in an isopropyl alcohol solution and distilled water with 5 wt% polymers (chitosan, Nafion, PVP, HPC, α-terpineol). It was observed that the chitosan and Nafion caused degradation of CuO, but Nafion formed a stable mixture when diluted. The modified electrodes were drop-casted and analyzed using cyclic voltammetry in 0.1 M KCl + 3 mM [Fe(CN)_6_]^3−/4−^ solution to determine the electrochemically active surface area (EASA). The results showed that α-terpineol formed agglomerates, while HPC created uneven distributions, resulting in poor stability. On the other hand, Nafion and PVP formed homogeneous layers, with PVP showing the highest EASA of 0.317 cm^2^. In phosphate-buffered saline (PBS), HPC and PVP demonstrated stable signals. Nafion remained the most stable in various electrolytes, making it suitable for sensing applications. Testing in 0.1 M NaOH revealed HPC instability, partial dissolution of PVP, and Cu ion reduction. The type of polymer used significantly impacts the performance of CuO sensors. Nafion and PVP show the most promise due to their stability and effective dispersion of CuO. Further optimization of polymer–CuO combinations is necessary for enhanced sensor functionality.

## 1. Introduction

So far, the fastest growing group of electrochemical sensors against various biomolecules are non-enzymatic receptors. In contrast to the most common enzyme-based sensors, they do not suffer from the immobilization problem, show enhanced sensitivity and selectivity, and are mostly unaffected by pH changes [1,2,3]. However, the critical step in their preparation is selecting the appropriate catalyst and electrode construction [4]. Their production process should be simple to develop, reproducible, and cost-effective. From the chemical vapor deposition (CVD) [5], electrospinning [6], and molecular imprinting [7], the drop-casting technique is the most common one and widely used [8,9,10]. The modification of the bare electrode surface using various materials comprising the interaction between the receptor and the matrices. Especially in the case matrices, the applied materials should fulfill some requirements such as adequate electrical conductivity, high specific surface area, and effective powder dispersity in whole volume [11,12]. Therefore, it is essential to match these two components properly.

Several polymers are widely used in drop-casting-modified electrochemical sensors due to their desirable properties, such as conductivity, mechanical stability, and biocompatibility. Some of the most popular polymers include polyaniline (PANI) [13], polyvinyl alcohol (PVA) [14], polyvinylpyrrolidone (PVP) [15,16], Nafion [15,17,18,19,20], or chitosan [12,21]. The polymers are chosen based on their specific properties that enhance the performance of the receptors’ activity, such as ease of film formation. However, the choice of polymer depends on the particular application, the nature of the detected substances, and the environment. 

Chitosan is used to immobilize biomolecules due to its presence of functional groups for further modification and biocompatibility [12,21,22]. Nafion is characterized by high ionic conductivity and chemical stability and is commonly used to facilitate ion exchange and stabilize electrode surfaces [18,20]. Due to its chemical stability, PVA is often used as a matrix for immobilizing biosensors [14], while PVP allows for good film-forming [16]. Their main advantages and structure are summarized in Table 1.

On the other hand, cellulose-based polymers (e.g., hydroxypropyl cellulose, HPC) may be an alternative due to bioeconomic demand. These polymers can be used as a scaffold for other materials due to the hydroxyl group’s reactivity, which is considered a bonding network [23]. Another worth noting substance is α-terpineol. Its ability to dissolve a wide range of materials makes it ideal for creating uniform and stable dispersions, which are essential for the drop-casting process in sensor fabrication [24,25]. However, their role in non-enzymatic sensors has not been widely studied.

**Table 1:** Characteristic of the chosen polymers.

Polymer	Structure	Charge	Solubility	Additional Information	Reference
chitosan	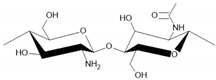	+	in acidic environment	biocompatible large contact surface area biodegradability	[12,21,22]
Nafion	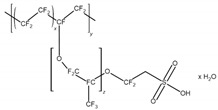	-	water ethanol	high ionic conductivity chemical stability film-forming ability	[15,17,18,19,20]
polyvinylpyrrolidone	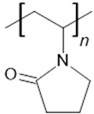	amphiphilic	water	film-forming ability biocompatibility biomolecules immobilization	[15,16]
hydroxypropyl cellulose	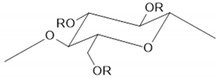	neutral	water, methanol, ethanol, isopropyl alcohol, acetone (polar organic solvents).	bonding network film-forming ability	[23,26]
α-terpineol	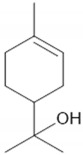	neutral	in water 2.4 g/L, benzene, acetone, alcohols, glycerol	biocompatibility non-toxicity film-forming ability	[24,25]

Chemical structures were drawn in the ChemSketch program version 2021.2.1.

The main component of the receptor is material dispersed in matrices. Copper oxide-based materials garnered significant interest in electrochemical sensors due to their high sensitivity, cost-effectiveness, and ease of synthesis [27,28,29,30]. However, challenges such as selectivity issues, surface fouling, and stability in harsh conditions must be addressed to leverage their potential fully [31,32,33]. It is noted that CuO on screen-printed electrodes can be used to detect carbohydrates (e.g., glucose, fructose) [34] and other biomolecules, such as hydrogen peroxide [35,36], dopamine [37,38], L-cysteine [39], L-tyrosine [40], acetaminophen, and caffeine [41]. In addition, copper ions are easily subjected to redox processes, making the drop-casting technique involve an appropriate choice of binding agent [42]. Advances in sensor design and fabrication techniques continue to mitigate some of these drawbacks, enhancing the performance and reliability of CuO-based electrochemical sensors.

In this work, we have studied the effect of the polymer used on the stability of the obtained film containing a copper(II) oxide-based sensing element. This research aimed to verify how we can influence the stability of a drop-casted receptor film by adjusting the binding substance’s physicochemical properties. Well-known polymers in the field of electrochemistry, such as Nafion or PVP, were compared with rarely applied hydroxypropyl cellulose and α-terpineol.

## 2. Materials and Methods

### 2.1. Materials

The electrodes were modified with commercially available copper(II) oxide powder (CuO, 99+%, CAS: 1317-38-0, Acros Organics, Waltham, MA, USA) without further processing. As a glue/polymer matrix, the following substances were used: chitosan (MW 100,000–300,000, CAS 9012-76-4, Acros Organics, Waltham, MA, USA), Nafion (D-520 dispersion, 5% *w*/*w* in water and 1-propanol, CAS 31175-20-9, Alfa Aesar, Haverhill, MA, USA), polyvinylpyrrolidone (MW 40,000, CAS 9003-39-8, Alfa Aesar, Haverhill, MA, USA), hydroxypropyl cellulose (MW~100,000, 20 mesh particle, 99%, CAS 9004-64-2, Sigma Aldrich, St. Louis, MO, USA), and α-terpineol (≥96%, CAS 10482-56-1, FW 154,25, Sigma Aldrich, St. Louis, MO, USA). The polymer solution was prepared by distilled water (DIW) and 2-propanol (99.7%, CAS 67-63-0, Avantor, Gliwice, Poland). A 1 M acetic acid solution (CAS 64-19-7, Avantor, Gliwice, Poland) was implemented to improve the chitosan’s solubility. For electrochemical characterization, K_4_[Fe(CN)_6_·3H_2_O (CAS 14459-95-1, Avantor, Gliwice, Poland), K_3_[Fe(CN)_6_)] (CAS 13746-66-2, Avantor, Gliwice, Poland), nominated 0.1 M sodium hydroxide solution (NaOH, CAS 1310-73-2, Avantor, Gliwice, Poland), and 0.1 M phosphate buffer solution (PBS, pH = 7.4, 448022B1, Reagecon, Clare, Ireland) were used.

### 2.2. Preparation of Modified Electrodes

Firstly, the polymer solutions were prepared by adding polymers (chitosan, Nafion, polyvinylpyrrolidone (PVP), hydroxypropyl cellulose (HPC), α-terpineol) to a solution of 2-propanol alcohol and distilled water (50/50 *v*/*v*) under constant stirring. To the solution containing chitosan, 15 mL of 1 M acetic acid was added to improve the solubility of chitosan.

A total of 5 mg of the powder was sonicated for 10 min in an appropriate solution, resulting in a 5 wt% concentration of CuO powder.

Afterward, 3 µL of the mix was drop-casted on a screen-printed electrode (SPE) with Ag as a reference and carbon as working (4 mm diameter) and counter electrodes (model C110, DropSens, Metrohm AG, Herisau, Switzerland). The electrodes were dried in the air at room temperature for 24 h. When not in use, they were stored in the refrigerator.

### 2.3. Characterization Techniques

The powder’s crystal structure was investigated using an X’Pert MPD diffractometer (Malvern Panalytical Ltd., Malvern, UK). The system operated in Bragg–Brentano geometry for X-ray diffraction analysis (XRD). Phase identification was performed using X’Pert HighScore Plus software (version 3.0.4) and the Powder Diffraction File (PDF-2). Scanning electron microscopy (SEM, ThermoFisher Scientific Apreo, Waltham, MA, USA) was used to perform the samples’ morphological characterization.

The dynamic light scattering (DLS) technique was used to measure particle dispersions’ hydrodynamic diameter (dh) in aqueous solutions. The electrophoretic light scattering (ELS) technique was used to characterize zeta and wall zeta potentials. The values were determined using Zetasizer Pro (Malvern Panalytical Ltd., Malvern, UK).

The images of the droplets deposited on the glass slide were analyzed using the 4 K High-Accuracy digital microscope Keyence VHX-7000 (Keyence, Mechelen, Belgium).

Modified screen-printed electrodes (SPE) were tested through the Interface 1010 TM Potentiostat/Galvanostat/ZRA (Gamry Instruments, Warminster, PA, USA).

The cyclic voltammetry (CV) technique was applied to determine the electrochemically active surface area (EASA) of the electrodes with 0.1 M KCl + 3 mM [Fe(CN)_6_]^3−/4−^ as an electrolyte. The samples’ electrochemical stability was determined by employing the phosphate-buffered saline (0.1 M PBS, pH = 7) and 0.1 M NaOH solution. All measurements were carried out at different scan rates (ν, from 10 to 2000 mVs^−1^).

Electrochemical impedance spectroscopy (EIS) tests were conducted in the three-electrode cell with modified SPE (total volume of 10 mL), Pt wire, and Ag/AgCl (3 M KCl) as working, counter, and reference electrodes. The frequency range of 10^−1^–10^6^ Hz and AC signal amplitude of 10 mV were applied. The measured data were analyzed with Echem AnalystTM Software version 7.10.0.12634. The model used to fit the experimental spectra consists of the resistors (R), constant phase elements (CPE), and Warburg element (W).

## 3. Results

Commercially available copper(II) oxide (CuO) powder was used as a reference material. This choice was driven by the ease of access to the powder, while copper oxide-based receptors are systems widely studied for use in glucose sensing [34].

However, the receptor’s form depends on the material’s synthesis procedure. So far, the most common technique is the drop-casting technique, whereas various polymers are used as the “glue.” The material’s stability and the created interface between the sensing element and the matrix polymer are responsible for the choice of the right compound. CuO presents several advantageous properties for application in electrochemical sensing, such as high sensitivity, catalytic activity, cost-effectiveness, versatility, and stability. On the other hand, materials need to be modified due to selectivity issues, surface fouling, reproducibility concerns, slower response times, and potential material degradation. Although the sensor design improvements can enhance the performance and reliability of CuO-based electrochemical sensors, they may affect the material distribution and interaction with the polymer (matrix).

### 3.1. CuO Receptor Characterization

**Figure 1:**
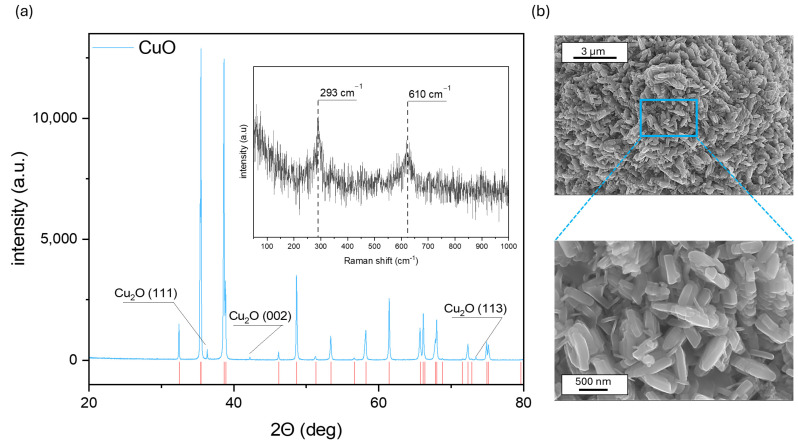
The XRD, Raman spectroscopy analysis (**a**), and SEM images (**b**) of the commercial copper oxide powder. The red vertical line in the XRD data corresponds with the CuO as a tenorite, C1c1 space group. Data were taken from ICSD #98-006-9757.

Figure 1 shows the XRD, Raman spectroscopy analysis, and microstructure images of the copper(II) oxide powder used. The powder’s phase composition analysis (Figure 1a) reveals the composition of CuO (tenorite, monoclinic, C1c1, ICSD #98-006-9757) with the addition of a small amount of Cu_2_O (cubic, Pn-3m, ICSD #98-026-1853). No additional peaks were recorded.

The vibrating bands reported for the crystalline structure of copper oxide (II) are observed at 293 (Ag) and 611 cm^−1^ (Bg) [43].

SEM examination (Figure 1b) of the powder morphology reveals the presence of a homogenous form of lath-shaped particles that are 590 nm long and 159 nm wide. The use of a powder with a rather specific shape will allow for (a) obtaining a mesoporous form immersed in the polymer, which will unambiguously affect the number of contacts between the matrix and the semiconductor, (b) creating a network of connections/transfer paths for electron carriers between grains, and (c) ensuring an adequate number of active sites at the receptor/electrolyte interface, even if the material will undergo agglomeration processes. Moreover, due to their lathe-like shape, these grains may exhibit planes with different surface energies, contributing to enhanced electrocatalytic activity.

**Figure 2:**
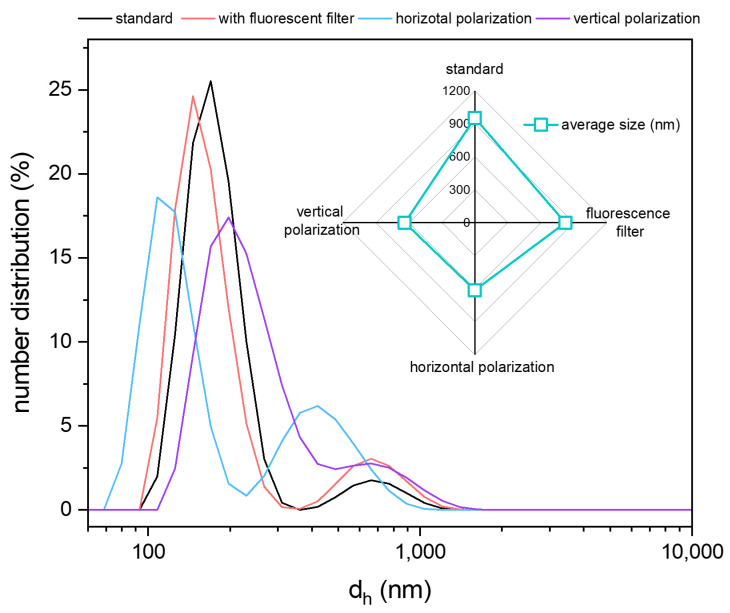
The dynamic light scattering (DLS) measurements of the hydrodynamic diameter (d_H_) CuO powder at various modes, including standard, with fluorescence filter, and with horizontal and vertical polarization, are estimated as a particle number distribution. The insert shows the average particle size calculated from each mode.

DLS measurements and zeta potential analysis may yield more information. The hydrodynamic diameter (d_H_) was evaluated using DLS in various modes, including standard, with fluorescence filter, and horizontal and vertical polarization. The results are shown in Figure 2. Despite the mode being applied at each scan, a two-modal distribution is visible, suggesting the presence of the two-grain fractions. This is in line with the SEM results, given the shape of the material. The average particle size was approximately 953.5 nm for standard analysis without any filter. The value is higher than those defined by scanning electron microscopy. The fluorescence filter does not improve these data. The recorded size is 821.4 nm. On the other hand, the polarization filter limits the collected light to the part with the same polarization as the input. Therefore, flare, or an artificial aspect from the cuvette, may be eliminated. The d_h_ decreased to 614.3 and 637.4 nm for horizontal and vertical filters, respectively. It shows a good correlation with SEM. 

### 3.2. Characterization of the Drop-Casted Layers

The degradation of the copper oxide materials with chitosan contact was observed during the preparation of the electrode pastes based on the polymer solution. The blue coloration indicated the passage of copper ions into the solution. The effect may be caused by a reaction between acetic acid, added to improve the solubility of chitosan by adjusting the pH, and cooper(II) oxide, which caused the formation of copper(II) acetate.

(1) $$CuO+2CH_{3}COOH\to Cu(CH_{3}COO{)}_{2}+H_{2}O$$

On the other hand, the reaction between the chitosan and CuO involves the formation of the coordination bonds between the amine groups from the polymer and Cu^2+^ ions, leading to the formation of stable Cu–amine complexes [44].

**Figure 3:**
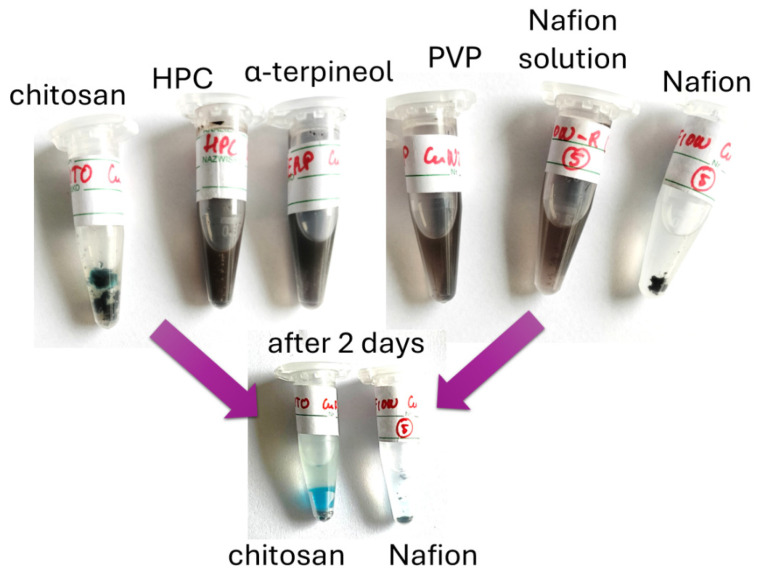
The images of the electrode pastes prepared with CuO powder and polymer solution.

A similar effect was observed for Nafion (Alfa Aesar, D-520 dispersion, 5% *w*/*w* in water and 1-propanol, USA). Figure 3 shows photos of the prepared pastes.

Therefore, an additional mixture of Nafion, distilled water, and isopropyl alcohol in the volume ratio 1:7:2 was prepared. In the case of the chitosan, the polymer was excluded from further investigation because no improvements were recorded for copper oxide powder, and decreasing the pH affected the powder’s stability.

**Figure 4:**
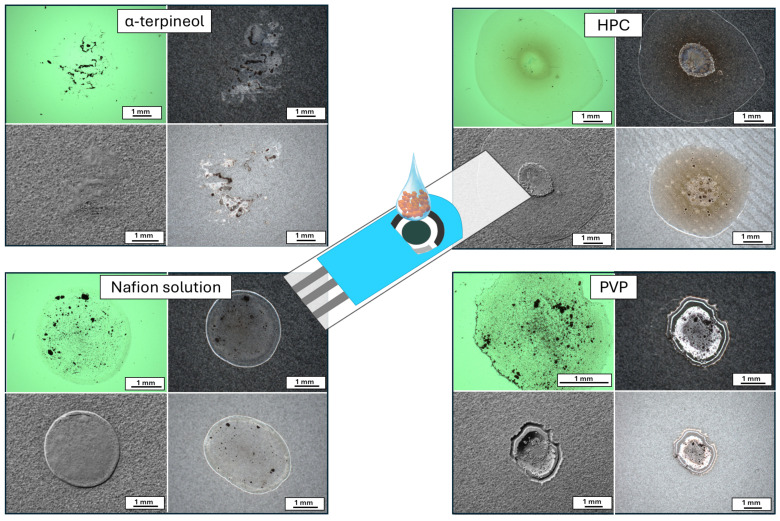
The optical microscope images of the paste drops prepared by the CuO powder and various polymer solutions. The photos were taken using (clockwise from top left) transmitted (green), reflected (black and light gray substrate) light and a high contrast function (gray images).

The copper oxide powder was deposited on the screen-printed carbon electrode using the drop-casting technique and analyzed for its stability in various electrolytes from its use as a receptor in non-enzymatic sensors. Images of the prepared electrode pasts are presented in Figure 4. Above all tested polymers, α-terpineol does not form a regular droplet and does not ensure an even distribution of the sensing material on the surface. Optical microscope images reveal receptor agglomerates. In the other three cases, the formed droplet is circular, but depending on the type of polymer, the material is differentially placed. In the case of HPC, the powder accumulates inside the circle. The resulting contact boundary between the electrolyte and the receptor is uneven, which can simultaneously affect the number of active sites and the size of the electrochemical active specific surface area. Only for the Nafion solution and PVP-based paste does the powder form a homogeneous layer, which can be attributed to a well-developed specific surface area. Moreover, due to the electrostatic forces interacting between the CuO and Nafion, which is considered the negatively charged polymer, it can be concluded that the grains are positively charged, guaranteeing their homogeneous distribution and affecting the formation of the charge transfer paths. On the other hand, PVP is inert. However, the chain structure effectively influences the trapped copper oxide particles. Based on the shape of the droplet and powder distribution, it may be assumed that for the α-terpineol, the carbon-printed electrode (SPE) signal may be recorded during the electrochemical measurements. Moreover, SPE can interfere with the signal recorded from the receptor, causing overlap or peak broadening/splitting.

### 3.3. Electrochemical Characterization of Modified Electrodes

**Figure 5:**
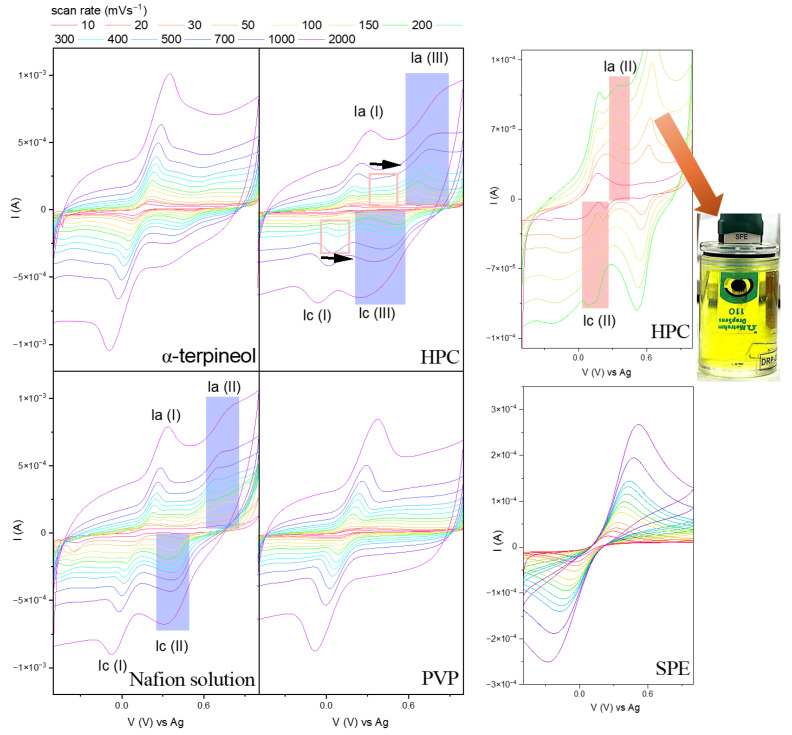
Current–voltage dependence for the modified carbon electrodes in the 0.1 M KCl + 3 mM [Fe(CN)_6_]^3−/4−^ with the image of the HPC-based SPE recorded in the middle of the measurements.

To see how the interaction between the polymer applied and the copper oxide affects their electrochemical activity, cyclic voltammetry was conducted in a chemically reversible ferrocyanide/ferricyanide solution (0.1 M KCl + 3 mM [Fe(CN)_6_]^3−/4−^). The results are presented in Figure 5.

The characteristic peak at the anodic (Ia) and cathodic (Ic) curves assigned to the electrolyte’s redox reaction is visible. Its intensity differs for each sample, depending on the powder homogeneity at the surface. Moreover, the redox peak currents (Ia/Ic) ratio was 1.09, 1.20, 1.01, and 1.46 for the α-terpineol, HPC, Nafion solution, and PVP, respectively, while for α-terpineol and Nafion-based pastes, the i_a_/i_c_ is approximately 1. This may suggest the [Fe(CN)_6_]^3−/4−^ redox reaction at the CuO surface is reversible, and the observed effect for HPC and PVP can be interpreted as quasi-reversible or irreversible.

Another way to define the reversibility of the process is the determination of the separation between the peak potentials at the anodic (*E_a_*) and cathodic (*E_c_*) curves [45]:

(2) $$∆E_{p}=E_{a}-E_{c}≅\frac{0.059}{n}$$

For a one-electron process, like a ferrocyanide/ferricyanide coupled system, the ΔE_p_ should equal 0.059 V. For the analyzed polymer/CuO receptors, the separation between peaks is 0.105, −0.084, 0.063, and 0.046 V. The results obtained align with those associated with the i_a_/i_c_ relationship. The Nafion solution/CuO system is the most favorable one.

**Figure 6:**
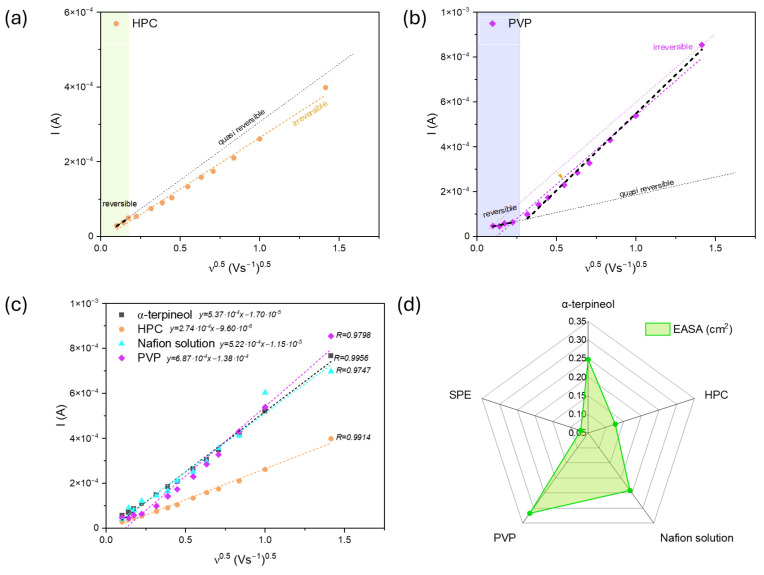
The plot of the anodic peak current as a function of the square root of the scan rate ν^0.5^ for (**a**) HPC, (**b**) PVP, and (**c**) summarized for all modified electrodes with (**d**) electrochemically active surface area of the SPE-modified electrodes.

The plot of the anodic peak current as a function of the square root of the scan rate ν^0.5^ for HPC and PVP (Figure 6a,b) revealed the complexity of the electrodes’ behaviors. The linearity range devoted to the reversibility of the redox reaction is narrow. Hydroxypropyl cellulose can create complexes by coordinating hydroxyl groups and Cu atoms and modifying the particle surface. Therefore, the irreversible mechanism is more probable (Figure 6a). On the other hand, PVP also should not be considered quasi-reversible (Figure 6b). Still, the linearity of the second range may suggest only changes in the mechanism (activation of the CuO surface), not the chemical composition of the CuO state. PVP contains carbonyl (C=O) groups in the pyrrolidone ring, which can coordinate with Cu atoms at the surface, prevent particle aggregation, and stabilize them. The activation of the formed electron transfer pathways affects copper oxide’s good response.

Based on the obtained results (Figure 5), the Randles–Ševčik Equation (2) was used to determine the electrochemically active surface area (EASA) **(**Figure 6c).

(3) $$I=2.69\cdot 10^{5}\cdot EASA\cdot D\cdot n^{2/3}\cdot C_{0}\cdot \nu^{0.5}$$

where *I* is the peak current (A), *D* is the diffusion coefficient of the [Fe(CN)6]^3−/4−^ redox species. Herein, 7.2·10^−6^ cm^2^s^−1^ *n* represents the number of the electrons involved in the reaction (*n* = 1), *C*_0_ is the concentration of the redox species (3 mM), and *ν* is the scan rate (Vs^−1^).

Despite using identical amounts of CuO, its dispersion in a given polymer differs and affects the formation of the interface between the powder and electrolyte. Implementing the HPC affects the number of available active sites (0.127 cm^2^). The PVP-based paste has the highest EASA, 0.317 cm^2^. The Nafion solution and α-terpineol paste are defined by the similar EASA, 0.241 and 0.248 cm^2^, respectively. Considering previous analyses on the distribution of the receptor in the polyether matrix, the size of the EASA for α-terpineol is surprising. It can be supposed that the irregularly agglomerated powder on the surface of the SPE can interact with the electrolyte like a solid electrode, and the system’s mesoporous nature provides adequate penetration for the electrolyte, thus increasing its EASA. Moreover, the previous conjecture related to the formation of charge transfer paths for Nafion is confirmed. Thus, the appropriate contact between the generated carrier during the redox reaction and the carbon substrate for the terminal-based paste is due to the thickness of the material agglomerates. For comparison, the SPE electrochemical active area is about 0.071 cm^2^, a few times lower than those for modified electrodes. The data are summarized in Figure 6d.

Notably, additional peaks attributed to the activity/reaction of the copper oxide materials with the electrolyte are present for the α-terpineol, HPC, and Nafion solution. However, those for α-terpineol are more highlighted for fast scans. The ferrocyanide/ferricyanide mixture is widely used to determine EASA due to the well-known diffusion coefficients of the reactants and rapid electron transfer. Additional peaks in CV curves can have different origins. Most often, for copper oxide-based materials [19,28], the effect of additional peaks is attributed to artifacts related to the migration of copper ions and their temporary change in the degree of oxidation as an effect of the reaction between [Fe(CN)_6_]^3−/4−^ with the electrode surface [46]. While their appearance does not affect the stability of the Nafion-based electrode, the HPC droplet is degraded. Its dissolution is observed during the measurements. On the other hand, the amphiphilic character of the PVP reduces interfacial tension and stabilizes emulsions or suspensions [47,48,49]. It can form thin, uniform films that modify surface properties, such as hydrophilicity, biocompatibility, and chemical resistance. This may reduce copper ion migration and limit the oxidation changes

**Figure 7:**
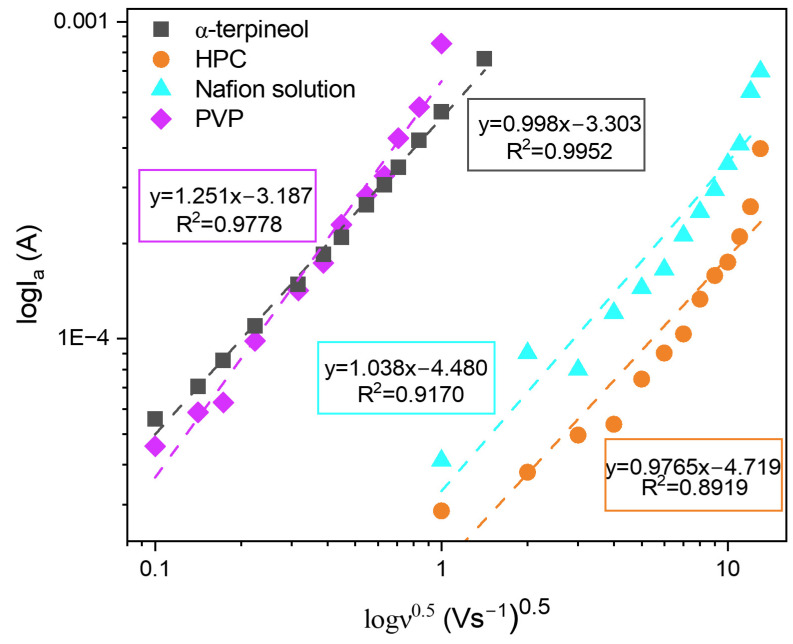
The plot of the logarithmic dependence of the square root of the scan rate (ν^0.5^) and recorded current in the 0.1 M KCl + 3 mM [Fe(CN)_6_]^3−/4−^ for all analyzed receptors.

Moreover, analyzing the correlation between the logarithmic dependence of the square root of the scan rate (ν^0.5^) and the oxidation peak current may reveal whether the process is adsorption or diffusion-controlled. As shown in Figure 7, the curves show good linearity despite the applied polymer. Moreover, the slope value in all cases is close to 1, which suggests that the observed redox process is surface-controlled.

Electrochemical impedance spectroscopy is the more sensitive technique to changes occurring at the electrode surface [50,51]. Thus, considering that the ferrocyanide/ferricyanide solution affects the HPC-based matrix dissolution, measurements were conducted for the rest of the polymers and the bare SPE [15,52]. The impedance spectra in the Bode and Nyquist configuration are shown in Figure 7. Due to the complex electrode structure, processes with different relaxation times were involved, which affected the impedance spectra according to various phenomena depending on the frequency range. Therefore, a wide frequency range was implemented to develop an accurate electric circuit model. Consequently, to fit the electrochemical impedance spectroscopy (EIS) data, a constant phase element (CPE) and a resistor with a Warburg element connected in perpendicular were employed. At high frequencies, the impedance is strongly related to the characteristics of the electrolyte. However, it is at low frequencies where the influence of diffusional mechanisms becomes truly significant, underscoring the importance of this factor in our research.

The difference between the SPE and the modified one is visible. The change in the character of the recorded curves indicates that the material immersed in the polymer completely covers the surface of the carbon electrode.

**Figure 8:**
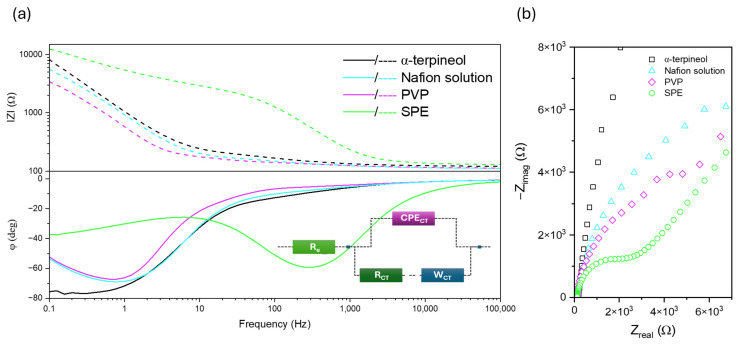
Impedance spectra in the Bode (**a**) and in the Nyquist (**b**) configuration for SPE and modified stripes in the 0.1 M KCl + 3 mM [Fe(CN)_6_]^3−/4−^ solution. The inset shows the equivalent circuit.

Figure 8b presents the analyzed electrodes’ impedance spectra in the Nyquist configuration. Based on the analysis of the impedance spectra and applied equivalent circuit, the charge transfer resistance (R_CT_), Warburg impedance modulus (W_CT_), and differential capacitance of the double layer (CPE_CT_) were evaluated for the SPE and receptor-modified by the Nafion solution. For the PVP and α-terpineol data, the characteristic for Warburg element slope at 45 deg is not visible. Therefore, the equivalent circuit modeling was made without W_CT_. The results are summarized in Table 2.

Firstly, for all modified electrodes, the α, defined as a deviation from capacitive behavior, is close to 0.8–1, corresponding to the Debbye capacitor. The polymer presence affects the R_CT_, which increases due to the formation of the dielectric shell [19]. This boosts the system’s capacitance by allowing the storage of electric charge. The charge transfer resistance increases in the case of polymer-modified SPE. The results suggest a higher ability to store charge, enhance the formation of an electric double layer at the electrode-electrolyte interface, and improve the sensor’s sensitivity.

While the [Fe(CN)_6_]^3−/4−^ environment is considered as a redox system for the electrochemical characterization of redox probe and neutral conditions, phosphate-buffered saline (PBS) and 0.1 M NaOH are close to the biological conditions required. Therefore, additional analyses were taken.

**Figure 9:**
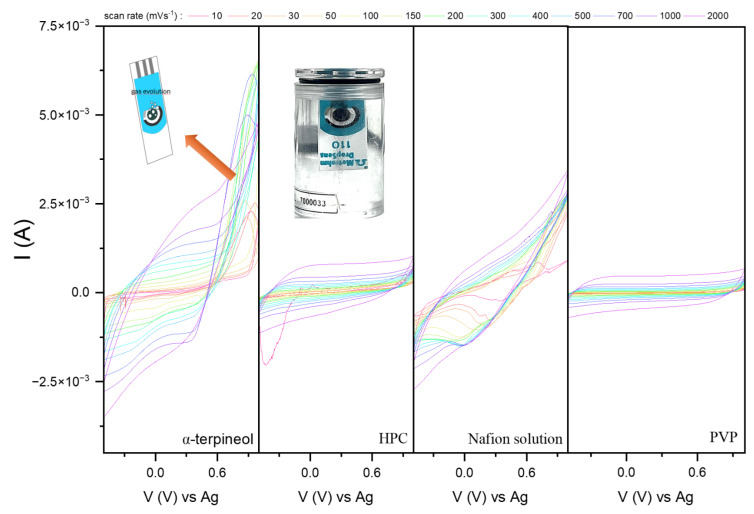
The cyclic voltammograms were recorded in the 0.1 M PBS electrolyte for the modified carbon electrodes with CuO pastes and various polymers. The increase in signal is accompanied by the appearance of bubbles on the surface of the electrode (arrow).

Figure 9 presents the cyclic voltammograms recorded in 0.1 M PBS electrolyte. PBS has a pH similar to that of human body fluids, which helps maintain the integrity of biological molecules and provides a consistent environment for electrochemical reactions. This compatibility is crucial when sensors are used in medical diagnostic or biological research. Moreover, this electrolyte should help stabilize the sensor’s surface, preventing degradation or fouling that could impair the sensor’s performance and the effects of the stable interface between the sensor and the analyte. As observed, the most stable signal, which can be assigned to the non-Faradaic contribution (no charge transfer), was recorded for the HPC and PVP pastes. It originates from forming a double layer at the electrode and monolayer surface, resulting in progressive charge storage. The difference between recorded CV curves may be affected by the properties of the polymers. Nafion is highly hydrophilic due to its sulfonic acid groups and can enhance the hydration layer around the electrode, affecting ion mobility and interfacial capacitance. Therefore, its ion exchange capabilities can modify the local environment and affect the non-Faradaic current. PVP, conversely, can influence the wettability and surface charge, impacting the adsorption and desorption processes of analytes. Its interaction with solvents and solutes can alter the mobility of ions in the vicinity of the electrode, impacting the capacitive behavior.

However, observing the electrode in operation revealed that HPC dissolves even in a non-ionic solvent. At the CV for α-terpineol, above a potential of about 0.9 V, a clear signal appears, associated with the gas formation on the electrode surface (insert photo). Surprisingly, the interface for the background is visible for the Nafion-based mixture. At the low scan rate, few oxidation peaks with corresponding reduction peaks and higher potential are present. Nafion is the most applied one in the case of the drop-cast electrodes for sensing, as it is non-active with contact with a wide range of electrolytes; therefore, it may relate to copper oxide [53].

**Figure 10:**
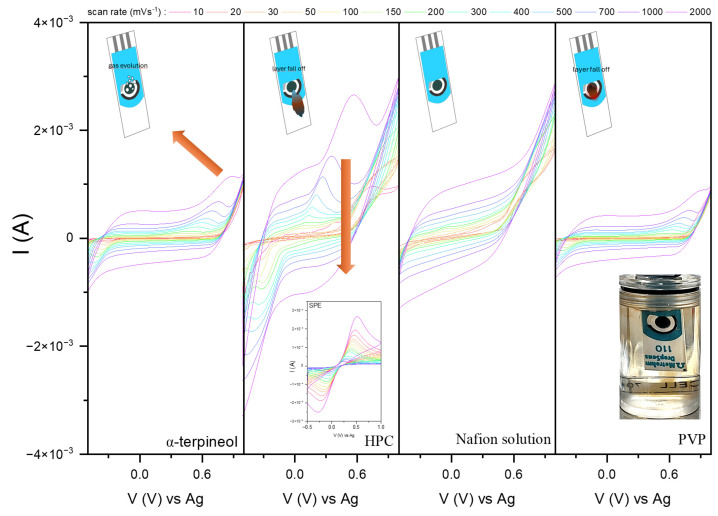
The cyclic voltammograms were recorded in the 0.1 M NaOH electrolyte for the modified screen-printed electrodes with CuO pastes and various polymers. The arrows indicate the source of the emerging signal.

Figure 10 shows the prepared electrodes’ stability measurements in a 0.1 M NaOH solution. Sodium hydroxide creates an alkaline environment that helps activate and maintain the surface. In redox reactions involving a non-enzymatic interaction of the receptor and biomarker, forming hydroxyl or oxygen bridges to facilitate electron carrier transfer is vital. Additionally, in some cases, the NaOH solution increases the ionic strength of the solution, improving its conductivity. The molar ionic conductivity of hydroxide ions in water and water-based solutions is approximately 198 Scm^2^mol^−1^.

Burke et al. highlighted the role of the pre-adsorbed hydrous oxides (OH_ads_) layer [54,55,56,57]. The model assumes that each surface is covered by a very thin outer layer of adatoms and/or incipient hydrous oxide, with a low bulk lattice coordination number, that may oxidize at low potentials and mediate the redox reaction:

(4) $$Me_{1}↔Me^{*}↔Me_{S}^{+}+e^{\prime }$$

(5) $$Me^{*}+yOH_{aq}^{-}↔[Me(OH{)}_{y}^{\left(y-x\right)}{]}_{ads}+xe^{\prime }$$

(6) $$adatoms\;or\;incipient\;hydrous\;oxide+RED\to Me^{*}+OX$$

where *Me*_1_ is a regular lattice atom, *Me_s_*^+^ are the ad-ions with the possibility to react directly with reductants (*RED*), and ads of the form [*Me*(*OH*)*_y_*^(*y*−*x*)^] are considered the adatoms and/or incipient hydrous oxide.

Satisfactory results were observed for all systems except HPC and PVP. Hydroxypropyl cellulose is unstable and affects film dropout during measurements. The exposed surface of the carbon electrode is active in the presence of hydroxyl ions, so redox signals associated with it begin to appear. In the case of PVP, two effects can be observed due to the reaction between the electrolyte and the receptor: (a) the reduction of copper ions from Cu^2+^ to Cu^1+^ (color changes from black to orange/red) and (b) the dissolution of the polymer matrix and slow release of the powder into the solution. Moreover, the formation of gaseous products affects the CV curves via the presence of additional peaks.

## 4. Discussion

The current study evaluates various polymers’ effects on the stability and performance of drop-casted receptor films containing copper oxide-based sensing elements. The goal is to enhance non-enzymatic electrochemical sensors by optimizing the binding polymers. The results indicate significant differences in performance and stability across the polymers tested, including chitosan, Nafion, polyvinylpyrrolidone (PVP), hydroxypropyl cellulose (HPC), and α-terpineol.

The analyzed polymers show good hydrophilic properties. PVP is due to the presence of the pyrrolidone rings capable of hydrogen bonding with water, and HPC is due to hydroxyl and hydroxypropyl groups [15,16,23]. Nafion shows an amphiphilic character, with significant hydrophilicity from its sulfonic acid groups, balanced by a hydrophobic backbone [17,18]. α-terpineol is considered moderately hydrophilic. It can form bonds with water molecules via its hydroxyl group. However, the presence of the hydrocarbon ring affects its hydrophobic nature [24]. CuO surfaces are typically strongly hydrophilic [58,59]. Therefore, this effect should not significantly impact the observed correlations.

Nafion and PVP emerged as the most promising candidates due to their superior electrochemical properties, stability, and effective material distribution. Surprisingly, while Nafion electrostatic stabilization is guaranteed by the interaction between sulfonic acid groups and material surfaces, PVP leads to the formation of colloids by bridging the particles [47,48,49]. The drop-casting technique with this polymer resulted in a uniform distribution of the copper oxide particles, contributing to an effective and stable interface between the receptor and the matrix. Moreover, the solution affects the chemical stability and ensures consistent sensor performance. On the other hand, PVP forms a consistent film and ensures uniform CuO particle distribution.

The zeta potential determines the material’s dispersion stability in the electrolyte. It is a key parameter in characterizing the surface charge properties of colloidal particles, including those found in suspensions of inorganic materials and polymers. Measuring zeta potential can provide valuable insights into these materials’ stability, compatibility, and interaction mechanisms. It is directly related to the electrostatic repulsion or attraction between suspended particles. It can reveal the surface charge characteristics of both the inorganic particles and the polymer. This is important for understanding their interactions, as like-charged surfaces typically repel each other, while oppositely charged surfaces may attract. By manipulating the surface charge (e.g., by adjusting the pH or adding surfactants), researchers can tailor the interaction between the inorganic material and polymer, optimizing the desired properties of the final composite material.

In systems where polymers are used as dispersing agents or stabilizers for inorganic particles, the zeta potential can provide insights into the nature and strength of the interaction at the interface. The analyzed copper(II) oxide shows a potential of about −24.41 mV, which correlates to the powder’s stable dispersion. Immersion of the particles in inert PVP does not change their properties; on the contrary, it ensures their uniform distribution between the polymer chains.

The polymer matrix effectively prevents particle aggregation and stabilizes the copper oxide particles, enhancing the sensor’s electrochemical response. It exhibits the highest EASA among the tested polymers, suggesting many active surface sites for electrochemical reactions.

Moreover, both polymers showed strong and consistent redox peaks, indicating effective electron transfer and stable electrochemical behavior. The I_a_/I_c_ ratios close to 1 for these polymers suggest a reversible redox reaction. They provided low charge transfer resistance (R_CT_) and high Warburg impedance (W_CT_), indicating efficient charge transfer and diffusion processes. The homogeneous layer formation contributed to their superior performance.

Conversely, chitosan, HPC, and α-terpineol showed significant drawbacks, including instability and uneven material distribution, adversely affecting sensor performance. Similarly to PVP, HPC and α-terpineol are neutral polymers. Therefore, their behavior should be comparable. However, the HPC demonstrated instability in both neutral and alkaline environments. It dissolved in the phosphate-buffered saline (PBS) and sodium hydroxide (NaOH) solutions, leading to the film’s degradation during electrochemical measurements. Additionally, the HPC-based films exhibited uneven material distribution, which affected the number of electrochemically active sites and the overall sensor performance. The more effective one seems to be α-terpineol. However, it forms a rather irregular droplet during the drop-casting process. This affects the uneven distribution of the sensing material, and the potential overlap of signals from the carbon electrode and the copper oxide receptor could complicate the interpretation of electrochemical measurements.

The receptor’s surface charge and electrostatic interactions with inert polymers should also be considered. In the case of chitosan, the formed suspension affects the material’s coagulation and degradation. To accurately predict receptor behavior, tests related to the surface charge (zeta potential) should be considered.

On the other hand, the sensor’s operating conditions also affect the stability of the matrix. Based on the results obtained, it can be concluded that, so far, Nafion provides both chemical stability and a relatively homogeneous distribution of the material on the surface of the modified electrode, which allows the obtaining of appropriate charge transfer paths.

Optimizing the concentration and combination of these polymers to further enhance the performance and reliability of copper oxide-based electrochemical sensors needs to be investigated. Exploring alternative polymers with tailored properties could provide new avenues for sensor development, addressing the limitations observed with the currently tested materials.

## 5. Conclusions

The electrodes were modified using commercially available copper oxide (CuO) powder with various polymers (chitosan, Nafion, PVP, HPC, α-terpineol).

The study evaluated the effects of various polymers on the stability and performance of drop-casted receptor films containing copper oxide (CuO)-based sensing elements. Each polymer demonstrated distinct advantages and disadvantages, influencing the sensor’s overall electrochemical behavior and stability.

When in contact with chitosan, CuO degraded and changed color to blue, indicating the release of copper ions. Due to material coagulation and degradation, it exhibits poor electrochemical performance.

It was shown that Nafion demonstrated excellent chemical stability across a range of electrolytes, including 0.1 M NaOH. Moreover, it forms a homogenous layer on the electrode surface, ensuring uniform distribution of the CuO particles. Nafion-based receptors exhibited superior electrochemical properties with strong and consistent redox peaks, indicating efficient electron transfer. It provides low charge transfer resistance (R_CT_) and high Warburg impedance (W_CT_), enhancing sensor performance. However, additional oxidation peaks at lower scan rates suggest specific interactions with CuO that might require further optimization.

Polyvinylpyrrolidone (PVP) produced the highest electrochemically active surface area (EASA) among the tested polymers (0.317 cm^2^), indicating many active sites for electrochemical reactions. It also formed a consistent and homogeneous film, ensuring a uniform distribution of CuO particles, and maintained stable redox peaks, indicating effective electron transfer. While PVP provided good stability in some conditions, it showed limited performance in 0.1 M NaOH, suggesting a need for further optimization in alkaline environments.

On the other hand, hydroxypropyl cellulose (HPC) demonstrated some electrochemical activity in neutral environments, with stable signals in 0.1 M PBS. However, it also exhibited significant instability in neutral and alkaline environments, dissolving in PBS and NaOH solutions. Noteworthy is the fact that the uneven material distribution leads to reduced electrochemically active sites and compromised sensor performance.

α-terpineol showed the potential for forming CuO-based films with moderate hydrophilic properties. The main drawback is the formation of irregular droplets during the drop-casting process, leading to uneven distribution of CuO on the electrode surface, and it caused gas formation on the electrode at higher potentials (~0.9 V), complicating electrochemical measurements and reducing stability.

Nafion is the most suitable for forming CuO-based receptors among the tested polymers. It provides the best balance of stability, material distribution, and electrochemical performance, making it the preferred choice for enhancing the performance and reliability of CuO-based electrochemical sensors.

## Figures and Tables

**Table 2:** Summary of the equivalent circuit parameters evaluated based on EIS measurements.

Parameter		SPE	α-Terpineol	Nafion Solution	PVP
R_e_	Ω	130.94	132.29	121.45	120.83
CPE_CT_	Ss^α^	2.06·10^−6^	1.95·10^−4^	2.31·10^−4^	2.47·10^−4^
α	-	0.9	0.8	0.9	0.8
R_CT_	Ω	2524.13	2.48·10^5^	13,106.98	16,562.93
W_CT_	Ω s^−0.5^	1.30·10^−4^		8.33·10^−4^	

## Data Availability

Dataset is available on request from the authors.
